# Extensive Spontaneous Coronary Artery Dissection Associated With Thrombosis: A Surgical Challenge

**DOI:** 10.1016/j.jaccas.2023.101752

**Published:** 2023-03-15

**Authors:** Katie E. O’Sullivan, Michael Z. Tong, Aaron J. Weiss, Faisal G. Bakaeen

**Affiliations:** Department of Thoracic and Cardiovascular Surgery, Heart, Vascular and Thoracic Institute, Cleveland Clinic, Cleveland, Ohio, USA

**Keywords:** spontaneous coronary artery dissection

## Abstract

Spontaneous coronary artery dissection is an uncommon cause of myocardial ischemia. Conservative management is the mainstay, although a few patients will require revascularization. We present a case of a 31-year-old woman whose extensive dissection necessitated coronary artery bypass grafting requiring an extended arteriotomy for excision of the thrombus and dissection flap. (**Level of Difficulty: Advanced.**)

## Central Illustration

**Figure:**
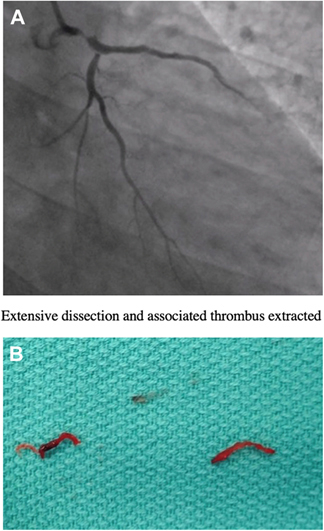

## History of Presentation

A 31-year-old woman presented to an outside hospital with severe central chest pain that had begun 7 days prior while she was physically exerting herself at the gym but intensified a couple of hours before arrival to the hospital. She was diaphoretic and looked unwell. She was in sinus tachycardia at 110 beats/min and normotensive.

**Learning Objectives**

• To understand the appropriate diagnosis and management of spontaneous coronary artery dissection.
• To learn techniques to successfully manage this condition surgically when required.

## Past Medical History

The patient’s medical history was unremarkable.

## Differential Diagnosis

The differential diagnosis included acute coronary syndrome, aortic dissection, pericarditis, myocarditis, pneumonia, esophageal spasm, and musculoskeletal pain.

## Investigations

**Figure 1:**
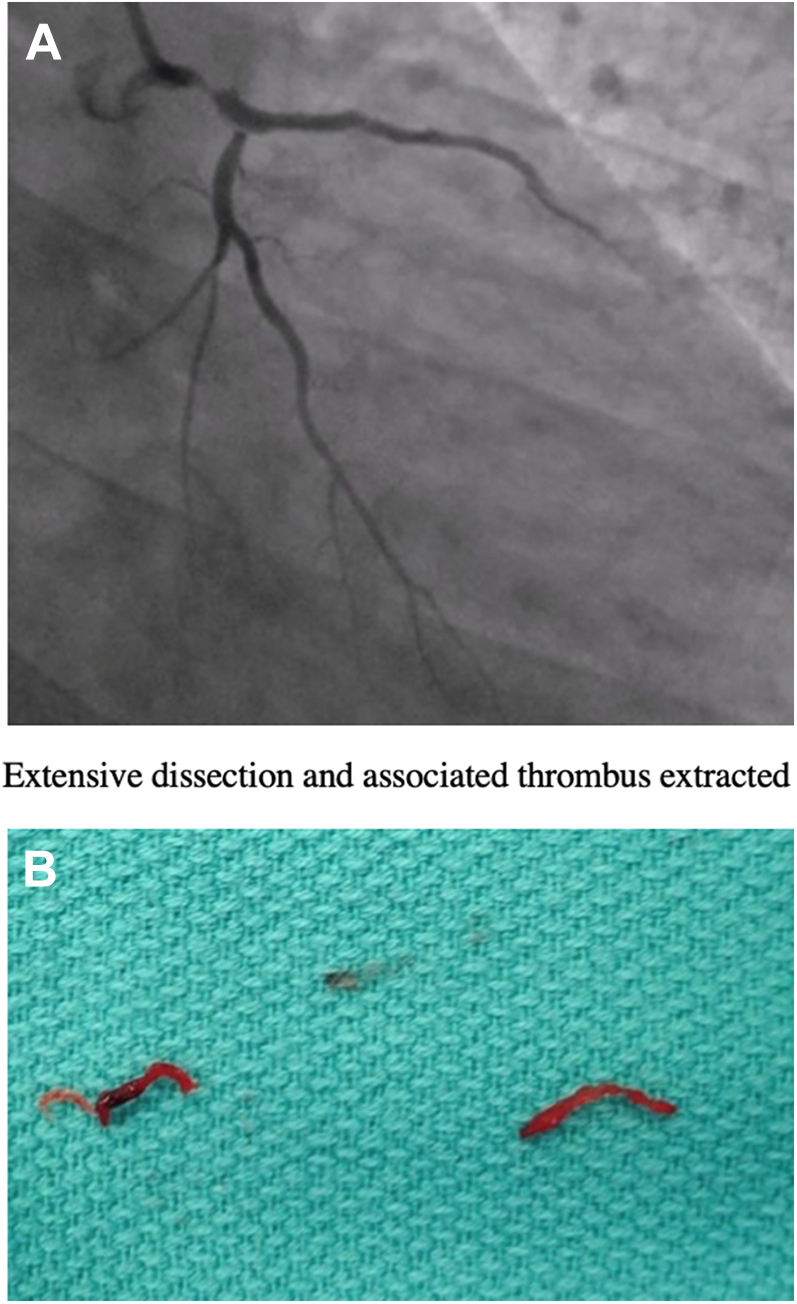
Preoperative Angiogram and Thrombus Extracted Intraoperatively Left main stem dissection with restricted flow in the left anterior descending and ramus **(A)** image of the extracted thrombus **(B).**

Electrocardiogram showed diffuse ST-segment depressions, and the patient’s troponin level was elevated at 4.770 ng/ml (reference range 0-0.29 ng/ml). She underwent coronary catheterization, which revealed a string-like dissected left main stem, with extension of the dissection into the ramus intermedius (RI) and left anterior descending (LAD) arteries (Figure 1A).

## Management

An intra-aortic balloon pump (IABP) was placed, and the patient was transferred to our hospital for further management. She continued to have angina despite ongoing medical management. On transthoracic echocardiography, the ejection fraction was 25%. Due to the patient’s ongoing chest pain, the decision was made to perform surgical revascularization with advanced mechanical circulatory support on standby (extracorporeal membrane oxygenation or Impella support [Abiomed]) given the severity of left ventricular dysfunction.

The left internal mammary artery and the greater saphenous vein were harvested. The patient was placed on cardiopulmonary bypass and after cross-clamping, antegrade and direct retrograde Buckberg cardioplegia were administered for cardioplegic arrest. Cold cardioplegia was administered every 15 minutes during the application of the cross-clamp, and a warm shot was used before removing the clamp. The RI vessel was discolored throughout its length. The vessel was opened distally and intracoronary thrombus extracted (Figure 1B). The arteriotomy was extended proximally and distally for several centimeters (≈5 cm) with clot evacuation and excision of intimal flap followed by flushing of the vessel using cardioplegia. The greater saphenous vein was anastomosed to the RI with 7-0 Prolene sutures (Ethicon) using a long anastomosis to incorporate the extended arteriotomy. Cardioplegia was delivered directly down the graft with excellent flow, and a proximal anastomosis was performed to the aorta. The LAD had a normal-appearing distal landing zone, and a left internal mammary artery to LAD anastomosis was performed. A warm shot of cardioplegia was administered and the clamp removed; the heart slowly regained activity on 4 mg/min of epinephrine. The anterior wall and the apex remained dyskinetic. Transit time flow was assessed and deemed satisfactory in both the LAD and RI grafts. The IABP was restarted, and the patient was weaned from cardiopulmonary bypass with a cardiac index of 2.9 L/min/m^2^. The cross- clamp and cardiopulmonary bypass times were 128 and 145 minutes, respectively.

**Figure 2:**
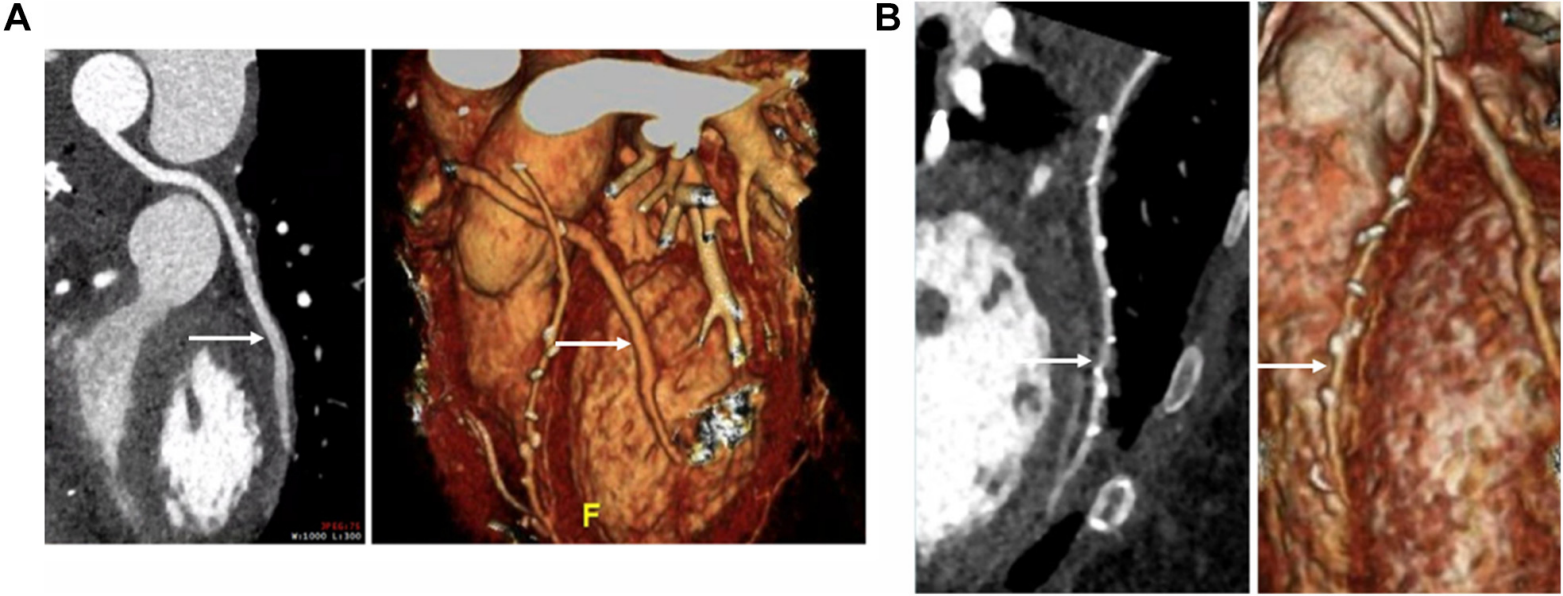
Postoperative Imaging **(A)** Three-dimensional computed tomography reconstruction of the patent saphenous vein to ramus intermedius graft (indicated by **white arrows**) with long distal anastomosis. **(B)** Three-dimensional computed tomography reconstruction showing the patent left internal mammary artery to distal left anterior descending graft (indicated by **white arrows**).

On postoperative day 2, the IABP was removed, and the patient was weaned off inotropic support. Computed tomography coronary angiogram was conducted on postoperative day 7 and showed patent grafts with a long distal anastomosis of the RI graft (Figure 2). Echocardiography before discharge showed that the ejection fraction was 30%. Immediately postoperatively, the patient was started on dual antiplatelet therapy and goal-directed medical therapy for heart failure. At 12 weeks’ follow-up, the patient reported a return to full activity without symptoms and an improved ejection fraction of 35%.

## Discussion

Spontaneous coronary artery dissection (SCAD) most often affects young women and has multiple triggers and predisposing conditions, emotional and physical stressors, connective tissue, and systemic inflammatory diseases, among others.^1^ Our patient described a history of physical stress that may have been a contributing factor.

There are three types of SCAD: I, evident arterial wall stain; II, diffuse stenosis of varying severity; and III, the subtype that mimics atherosclerosis.^2^ Recently published Spanish registry data revealed that percutaneous coronary intervention was required in ∼22% of SCAD cases, often with more severe lesions on presentation.^1^ A paucity of patients (≤8%) require coronary artery bypass grafting.^3^ The decision to revascularize depends on the patients’ clinical presentation and anatomy. Those patients requiring revascularization who are unsuitable for percutaneous coronary intervention pose several surgical challenges, particularly when the dissection extends into the distal coronary segments and in the context of extensive myocardial injury or infarction.^4^

In our patient, to effectively treat the dissection and intraluminal hematoma, we performed an extended arteriotomy. Although a standard bypass graft may be adequate for dissections limited to proximal portions of the coronary vessel, here nearly the entire RI was dissected and thrombosed. Furthermore, the patient was still having chest pain entering the operating room, and we therefore believed the myocardium could still be salvaged with revascularization. The extended arteriotomy allowed for en bloc excision of the thrombus and dissection flap. We planned to have advanced mechanical circulatory support on standby if unable to wean from cardiopulmonary bypass.

Interestingly, poor patency rates have been reported for bypass grafts in the context of coronary dissection. This finding is likely due to remodeling of the dissected vessel wall and resorption of intramural hematoma, with resultant competitive flow from the native vessel, and thus is often not clinically significant for the patient.^5^

## Conclusions

Our case demonstrates a useful surgical approach in patients with coronary dissections that extend into distal segments of the vessels and in patients with thrombosed coronaries.

## Funding Support and Author Disclosures

The authors have reported that they have no relationships relevant to the contents of this paper to disclose.
